# Accuracy of Intraoral Scanner for Recording Completely Edentulous Arches—A Systematic Review

**DOI:** 10.3390/dj11100241

**Published:** 2023-10-18

**Authors:** Gunjan Srivastava, Subrat Kumar Padhiary, Neeta Mohanty, Pedro Molinero-Mourelle, Najla Chebib

**Affiliations:** 1 Department of Prosthodontics, Institute of Dental Sciences, Siksha ‘O’ Anusandhan, Deemed To Be University, Bhubaneswar 751003, Odisha, India; gunjansrivastava@soa.ac.in; 2 Department of Oral and Maxillofacial Surgery, Institute of Dental Sciences, Siksha ‘O’ Anusandhan, Deemed To Be University, Bhubaneswar 751003, Odisha, India; subratpadhiary@soa.ac.in; 3 Department of Oral Pathology and Microbiology, Institute of Dental Sciences, Siksha ‘O’ Anusandhan, Deemed To Be University, Bhubaneswar 751003, Odisha, India; neetamohanty@soa.ac.in; 4 Department of Reconstructive Dentistry and Gerodontology, School of Dental Medicine, University of Bern, 3010 Bern, Switzerland; pedro.molineromourelle@unibe.ch; 5 Division of Gerodontology and Removable Prosthodontics, University Clinics of Dental Medicine, University of Geneva, 1205 Geneva, Switzerland

**Keywords:** intraoral scanner, conventional impression, edentulous jaws, removable prosthesis, complete denture

## Abstract

Scanning edentulous arches during complete denture fabrication is a crucial step; however, the quality of the resulting digital scan is still questionable. The purpose of this study is to systematically review studies (both clinical and in vitro) and determine whether intraoral scanners have clinically acceptable accuracy when recording completely edentulous arches for the fabrication of removable complete dentures. An electronic search in medical databases like PubMed, Scopus, and Web of Science (WOS), using a combination of relevant keywords, retrieved 334 articles. After full-text evaluation, twelve articles fulfilled the inclusion criteria for this review (eight clinical studies and four in vitro studies). A quality analysis of the included studies was carried out using the QUADAS-2 tool. The accuracy values varied between different intraoral scanners. Different regions of the edentulous arches showed differences in trueness and precision values in both in vitro and clinical studies. Peripheral borders, the inner seal, and poorly traceable structures like the soft palate showed maximum discrepancies. The accuracy of intraoral scanners in recording clear anatomic landmarks like hard tissues with attached mucosa was comparable to conventional edentulous arch impressions. However, higher discrepancies were recorded when digitizing mobile and poorly traceable structures. Intraoral scanners can be used to digitize denture-bearing areas, but the interpretation of the peripheral border and the soft palate should be carefully carried out.

## 1. Introduction

The optimum function of a complete denture depends on the tight fit of the intaglio surface of the complete denture and the peripheral seal area, with the cohesive forces of saliva adding to the retention [1]. Different impression-making techniques rely on these philosophies to record the denture-bearing area. The mucostatic concept is based on Pascal’s law of fluid dynamics [2]. It suggests that denture retention relies on the intimate contact of the denture base on the residual ridge [3]. The mucodynamic technique records the peripheral tissues in a functional state, thus resulting in a peripheral seal that is not disturbed during muscle movement. Impression-making for complete denture fabrication aims to achieve an accurate replica of the hard and soft tissues of the denture-bearing area [4,5]. The gold standard for master impressions of edentulous jaws is two-stage impression-making with a low-fusing thermoplastic material used for border molding followed by an impression with zinc oxide eugenol impression paste or a silicone material [6]. Clinicians have attempted to simplify this step and use elastomeric impression materials like polyvinyl siloxane (PVS) for one-stage border molding and the final impression [7]. Currently, the intraoral scanner (IOS) is a standard tool for the impression-making procedure for fixed and implant-supported prostheses with similar or better accuracy [8,9,10]. Capturing the anatomy of an edentulous jaw with movable, pliable mucosa, a complex texture, and variable anatomy is a challenging dental procedure [11].

Nevertheless, scanning reduces patient discomfort as no impression material is placed on the tissues, and the tissues are not deformed during the impression. Moreover, it allows for the easy transfer of information to the laboratory technicians and the archiving of data related to prosthesis fabrication [12]. The denture-bearing areas are smooth, shiny, and devoid of clear anatomic landmarks. These can induce errors during the stitching process [13,14]. Some authors suggested adding landmarks to the mucosa to help the IOS stitch the images [15,16]. Reports on the fabrication of complete dentures using the IOS have suggested retraction of lips, cheek, and tongue while performing the intraoral scan. The IOS can record the tissues in a mucostatic condition; however, some difficulties can arise when recording the functional depth of the vestibule, as the IOS tip size may hinder access around the tuberosity in the posterior maxilla [17]. The feasibility of the digital workflow, from the intraoral scanner to the fabrication of a completely retentive and functionally effective denture, was illustrated in case reports [18,19]. 

The capabilities of the IOS in scanning denture-bearing areas were previously investigated; however, the resulting accuracy of these scans is still inconclusive. This systematic review aims to determine whether intraoral scanners have clinically acceptable accuracy when recording completely edentulous arches to fabricate removable complete dentures.

## 2. Material and Methods

The protocol for this systematic review was registered in the International Prospective Register of Systematic Reviews (PROSPERO) with the registration number CRD42021289821. It was conducted following the Preferred Reporting Items for Systematic Reviews and Meta-Analyses (PRISMA) guidelines.

The PICO format for the current study is as follows: (P) the population is a completely edentulous patient, (I) the intervention is an impression made using an IOS, (C) the comparison is a conventional impression technique used for the completely edentulous arch, and (O) the outcome assessed is accuracy in the form of trueness and precision. 

The inclusion criteria consisted of studies comparing the accuracy of intraoral scanners for recording completely edentulous arches. Studies that compared digital scans with conventional impressions, in in vitro or in clinical studies, were included. The studies were clinical nonrandomized studies. There were no restrictions on the publication date or languages of the included studies. Studies that included complete arch implant impressions, case reports/series, and studies where accuracy was not determined were excluded. Review articles were also excluded.

### 2.1. Study Selection

**Figure 1:**
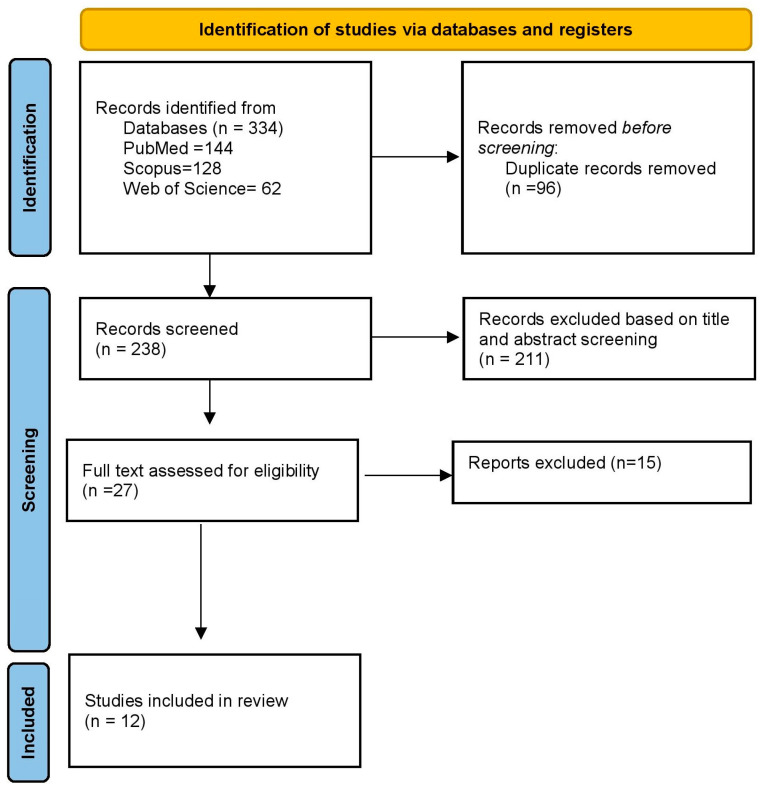
Prisma flowchart for study selection.

Two independent reviewers (G.S., S.P.) performed the initial search in online databases like PubMed, Scopus, and Web of Science using the relevant keywords in different combinations. Table 1 depicts the search strategy. A preliminary electronic search retrieved 334 articles, which were imported into a citation manager (ZOTERO, Corporation for Digital Scholarship, Fairfax, VA, USA, version 5.0), and duplicates were deleted. The title and abstract were screened, and articles were included in the review if they fulfilled the eligibility criteria (Figure 1). Any disagreement in the inclusion of articles was resolved by a third reviewer (N.C.).

### 2.2. Risk of Bias Assessment

The risk of bias for all included studies was evaluated by two independent reviewers using the Quality Assessment Tool for Diagnostic Accuracy Studies-2 (QUADAS-2). This tool is specifically designed to assess the quality of primary diagnostic accuracy studies. It consists of four domains that cover patient selection, index test, reference standard, and flow and timing. The relevant questions from QUADAS-2 were applied to the eligible studies, and a scoring system was used, where a score of 1 was assigned for “yes” answers and a score of 0 for “no” or “unclear” answers. The maximum score achievable was 13, indicating a low risk of bias (Table 2).

### 2.3. Data Extraction

The following outcome variables were extracted from the included studies: year of publication, type of study, country, sample size, type of intraoral scanner, experimental method, accuracy evaluation method, and mean deviation from the conventional impression expressed in mm (precision or trueness).

## 3. Results

From the electronic search in different databases, 334 articles were identified (PubMed *n* = 144, Scopus *n* = 128, Web of Science *n* = 62). After removing the duplicates, 238 articles were screened for eligibility. Based on the title and abstract screening, 211 articles were excluded. Further, 27 full texts were assessed, and based on the inclusion and exclusion criteria, 12 studies were included in the review (Figure 1): 8 clinical [20,21,22,23,24,25,26,27] and 4 in vitro studies [13,28,29,30]. Altogether, 126 patients were included in the clinical studies, and the total sample size was 50 for in vitro studies. The geographic distribution shows that the included studies were limited to a few countries. Table 3 depicts the list of excluded articles with reasons for exclusion. 

### 3.1. Risk of Bias Assessment

Based on Table 2, all the studies included in the assessment demonstrated a low risk of bias. In terms of the patient selection domain, all studies except one [23] provided clear descriptions. For the flow and timing domain, it was considered unclear when the timelapse between the reference and test methods was not clearly explained in studies [22,23,26]. In cases where intermediate test results were reported [23,30] and withdrawal from the study was clearly explained [20,24,26], these factors were marked as “yes” and scored as 1. However, it is important to note that the withdrawal process and timelapse factor between the reference and test method were not taken into consideration for in vitro studies [13,28,29,30].

### 3.2. Characteristics of Clinical Studies

Among the evaluated intraoral scanners, the Trios (TRIOS; 3Shape A/S) was the most used, with six clinical studies [20,21,23,25,26,27] utilizing it. One study [24] employed Lava COS (3M ESPE) and True Definition (TD) (3M ESPE), while another study [22] used CS3500 Carestream. Table 4 provides an overview of the outcomes extracted from these clinical studies. Four out of the eight studies focused solely on the maxilla [20,21,25,26], while the remaining four assessed both the maxilla and the mandible [22,23,24,27]. Four studies described root mean square (RMS) values, which consider both negative and positive deviations [21,25,26,27], and four studies calculated mean differences [20,22,23,24]. The trueness values for the entire surface of the scans ranged from 0.5 to 1 mm in most of the included studies [21,24,27].

Due to the heterogeneity of outcome variables reported in the studies, a meta-analysis could not be conducted. Some studies reported mean average deviations, while others reported root mean square deviations. The accuracy of the intraoral scan was primarily evaluated by superimposing it against a 3D model obtained from a lab scanner in most studies [20,21,22,26,27]. One study evaluated accuracy by superimposing the intraoral scan against a 3D model obtained from an IOS [23]. In another study, conventional impressions and the resulting stone casts were digitized and superimposed over the intraoral scan [24]. Kalberer et al. used a custom-made 3D comparison software and selected three reference points to superimpose the impression [25].

The superimposition of virtual models was performed using Geomagic, a 3D aligning software, with best-fit alignment. However, it is worth noting that different versions of the same software were utilized in six separate studies. In one study [20], 3D Reshaper was used as the aligning software, while another study [25] employed custom-made comparison software. Regarding the scanning pattern described by Hack et al., the maxillary arch scanning initiated from the right tuberosity and followed a zigzag path, concluding at the anterior part. For the mandibular arch, scanning began at the right retromolar pad area, following a zigzag path towards the opposite side. Additional scans were taken of the vestibule and sublingual regions. This scanning pattern ensured sufficient overlap and capture of adjacent structures [24].

Zarone et al. described the three scanning techniques. The buccopalatal technique (BP) first scans the edentulous ridge top starting from the left maxillary tuberosity, then moving along the buccal aspect and with an anticlockwise movement along the palatal surface and completing at the midline of the palate; in the S-shaped technique, the scanning started from the palatal side of the left maxillary tuberosity by moving the scanner tip with alternate palatobuccal and buccopalatal S-shaped movements along the ridge, from the left to the right side; finally, the area along the palatal midline was recorded. In the palatobuccal technique, the scanning advanced from the left maxillary tuberosity along the top of the ridge, ending at the right side, then covered the palatal side and terminated on the buccal side [30].

### 3.3. Characteristics of In Vitro Studies

The IOS used were Trios3 (TRIOS; 3Shape A/S) [28,29,30] and Cerec Omnicam/Cerec Bluecam (Dentsp Superimposition against 3D model obtained from lab scanner.ly Sirona) in three studies [13,28,29]; Itero (Align Technology) [13,28] and Planmeca Emerald (PE) (Planmeca) [28,29] each in two studies; and Lava COS (3M ESPE) [13] and True Definition (TD) (3M ESPE) [29] in one study each. Table 5 reports the outcomes of the in vitro evaluations with mean trueness ranging from 44.1 to 591.8 μm in the in vitro study carried out by Patzelt et al. [13] and an improvement of trueness value in subsequent studies with average trueness values of 127.2 ± 60 μm reported by Braian and Wennerberg [28] and 64.98 ± 23 μm reported by Zarone et al. [30].

Two studies evaluated the accuracy by superimposing an intraoral scan against a 3D model obtained from a laboratory scanner [13,30]. Another study measured the distance between markers in an edentulous arch and comparison with the reference data [28], and Osnes et al. [29] superimposed the 3D data within each group to assess precision.

## 4. Discussion

The included studies indicate that the accuracy of an edentulous mucosa scan depends on soft tissue features such as flexibility, mobility, and dimension [21,24,27], as well as on IOS system characteristics, including wand size and width, and the scanning strategy [23,24,30]. Lo Russo et al. described the workflow for scanning edentulous maxillary and mandibular arches and the simultaneous recording of maxillomandibular relations [45].

Zarone et al. [30] assessed the influence of scanning strategy on the accuracy of the digital scan. They found that the buccopalatal technique showed better mean values for trueness and precision than the palatobuccal technique. The s-shaped scan is recommended to reduce errors where scanning starts from the left maxillary tuberosity, and the scanner tip is moved alternatively between palatobuccal and buccopalatal in S-shaped movements along the ridge, ensuring enough overlap with previously scanned areas. Patzelt et al. [13] were the first to publish on intraoral scanning of the edentulous jaws. They suggested some enhancements in intraoral scanners before using them in clinical practice.

Most of the included studies used the best-fit algorithm software that stitches together the images acquired by the IOS. The studies that assessed the trueness and precision of the digital scans employed different aligning software, some of which were custom-made for the specific study [25,29]. There were variations in the required aligning software and the methodology used to match the scans. Some studies utilized the conventional impression scan as a comparison reference [21], while other researchers used the digital scan as a reference for the software [23].

The assessment of best-fit alignment and color-coded comparisons of the maxilla revealed that maximum deviations were present at the soft palate, peripheral borders, or the inner seal in an intraoral scan. The highest variation was found in sublingual areas and the vestibule in the mandibular arch. The authors reported that lips and cheeks must be considerably retracted during the scanning procedure [21,24], which could be the reason for more discrepancies in peripheral areas. The clinically acceptable discrepancy value for removable prosthesis accuracy should match the compressibility of the tissues to avoid the creation of friction areas, sore spots, and the need for additional denture adjustment sessions. Among the included studies, accuracy values ranged from 300 to 500 µm [22,24,29], which is close to the mean value for the compressibility of tissues [46]. Lo Russo et al. [23] found that the mean distance between full scans (−0.19 ± 0.18 mm) before trimming was reduced to (−0.02 ± 0.05 mm) after trimming. They suggested that when the intraoral scan is appropriately trimmed, it reduces the nonmatching peripheral areas, resulting in a marked improvement in the overall alignment accuracy. They also suggested that IOS is a mucostatic technique and any apical position of conventional impression is due to compression. 

Lo Russo et al. [23] found that trimming peripheral areas from both files, i.e., IOS and conventional impression, resulting from mobile tissue stretching and compression improved alignment and accuracy. Chebib et al. [26] suggested that trimming the peripheral borders of conventional impressions to a similar extent to the intraoral scan improved the accuracy value, resulting in statistically similar intraoral scans and conventional impressions.

In a dimensional measurement study on the mandibular arch, Braian and Wennerberg calculated the distance between five markers in pairwise comparison and cross-arch comparison. They reported that complete arch scans had low precision, although short distances of about 22 µm presented better accuracy. The study showed an inversely proportional relation between scan distance and 3D data accuracy [28].

Osnes et al. [29] described that Planmeca and Dental Wings produced the most considerable mean surface deviations over the entire surface. However, these values were below the threshold value of 300 µm, whereas Aadva showed the lowest and most consistent values. Trios, Aadva, Omnicam, and TDS all produced clinically acceptable scans. Jung et al. [22] assessed the accuracy of IOS in only the supporting tissues in the maxillary and mandibular arches. The authors suggested that better results may be obtained with a scanner with customized tips for targeting soft tissues. 

Using custom-made software, Kalberer et al. [25] evaluated the vertical and horizontal discrepancies. No significant difference was found between the digital scan and the polyvinyl-siloxane impression. In the anterior region, the digital scan was less accurate than the polyvinyl siloxane. This might be due to a change in the scanning direction. The polyvinyl siloxane was significantly more accurate than the digital scan at the inner seal and about 2 mm from the border molded impression.

The influence of the operator’s experience on scan accuracy and operating time was studied by Schimmel and Deferm et al., indicating that the operator’s experience and shorter scanning time improved the accuracy of the digital scan [32,35]. A fully edentulous scan for the mandibular arch revealed significantly superior trueness, and an edentulous scan for the maxillary arch provided significantly better precision by the inexperienced operator. However, these studies were conducted on models of edentulous arches that do not replicate the exact intraoral condition, such as the presence of saliva and mobile tissues that can change impression accuracy [32,35]. 

With technological advances, new-generation IOSs show higher accuracy and shorter scan times. Software and hardware updates over the years have been implemented to improve data acquisition. Although the accuracy of an IOS depends on the precision of each image captured, it also relies on the accuracy of superimposition, which is determined by the mathematical models and algorithms used to reconstruct the image [47]. 

The limitations of this study include clinical studies comparing digital scan data to the conventional impression with constraints. Conventional impressions needed to be digitized by a laboratory scanner, which could introduce some known or unknown distortion. Additionally, in some studies, the cast was made and digitized, leading to variations in results. Nonetheless, this method was a valid approach to revealing the shortcomings of an evolving technology and providing recommendations to clinicians. Most of the included studies could not compare the effect of the digital scan on the retention and stability of complete dentures. However, one of the included studies by Chebib et al. [26] assessed the difference in accuracy between the conventional and digital impressions and the fit and retention of complete denture bases fabricated by the 3D printing and milling techniques. The authors found a discrepancy of 0.45 mm between the scan and the definitive cast. However, this slight discrepancy on the entire surface of the scan resulted in a two- to three-times reduction in retention for the resulting denture base. 

## 5. Conclusions

Within the limitations of the current systematic review, intraoral scanners provide clinically acceptable digital scans. However, some improvement should be implemented when recording the mobile mucosa surfaces. Studies should standardize the quantification methods of discrepancies and explore the clinical significance of those deviations on the clinical fit and comfort of resulting complete dental prosthesis. Digital intraoral scanning is not recommended when facing unfavorable ridge anatomy and when denture retention may require the compression of the tissues.

When scanning the vestibule, the entire area should be scanned in one shot, as rescanning later to capture the missed area may result in error. Additionally, the intraoral scanner can be used to digitize edentulous arches, although case selection is still required. 

## Figures and Tables

**Table 1:** Search strategy.

Focused Question	In Fully Edentulous Patients, What Is the Accuracy of IOS Compared to Conventional Impression Techniques in the Form of Trueness and Precision for Complete Dentures?	
**PICO**	**Population**	Fully edentulous patient (((edentulous arch) OR (edentulous jaw [MeSH Terms])) OR (jaw edentulous [MeSH Terms])) OR (edentulous ridge).
	**Intervention**	Edentulous impression with an IOS ((((digital impression) OR (intraoral scanner)) OR (optical impression)) OR (digital scan)) OR (intraoral scan).
	**Comparison**	Conventional impression techniques (((dental impression technique [MeSH Terms]) OR (conventional impression)) OR (conventional technique)) OR (elastomers, silicone [MeSH Terms]).
	**Outcome**	Accuracy in the form of trueness and precision for complete dentures ((((data accuracy [MeSH Terms]) OR (accuracy)) OR (precision)) OR (trueness)) OR (3D comparison).
**Search Strategy**	**Pubmed**	(((((edentulous arch) OR (edentulous jaw[MeSH Terms])) OR (jaw edentulous[MeSH Terms])) OR (edentulous ridge)) AND (((((digital impression) OR (intraoral scanner)) OR (optical impression)) OR (digital scan)) OR (intraoral scan))) AND ((((dental impression technique[MeSH Terms]) OR (conventional impression)) OR (conventional technique)) OR (elastomers, silicone[MeSH Terms]))) AND (((((data accuracy[MeSH Terms]) OR (accuracy)) OR (precision)) OR (trueness)) OR (3D comparison)).
**Database Search**	MEDLINE (PubMed), Scopus, and Web of Science (WOS).	

**Table 2:** Qualitative analysis of included studies by Quality Assessment Tool for Diagnostic Accuracy Studies-2 (QUADAS-2).

	Criteria	D’Arienzo [20]	Chebib [21]	Jung [22]	Lo Russo [23]	Hack [24]	Kalberer [25]	Chebib [26]	Alhamad [27]	Patzelt [13]	Brian [28]	Osnes [29]	Zarone [30]
1.	Was the range of the edentulous mucosa representative of what will be identified clinically?	1	1	1	1	1	1	1	1	1	1	1	1
2.	Were criteria for selection clearly described?	1	1	1	0	1	1	1	1	1	0	1	0
3.	Control method likely to correctly classify the target condition	1	1	1	1	1	1	1	1	1	1	1	1
4.	The timelapse between the reference method and test method is short enough so the target tissue does not change	1	1	0	0	1	1	0	1	0	0	0	0
5.	Did the whole sample receive the verification?	1	0	1	0	1	0	1	1	1	1	1	1
6.	Edentulous mucosa received the same control method regardless of the test method	1	1	1	1	1	1	1	1	1	1	1	1
7.	Was the control method independent of the test method?	1	1	1	1	1	1	1	1	1	1	1	1
8.	Test method execution described in detail	1	1	1	1	1	1	1	1	1	1	1	1
9.	Execution of the control method described in detail	1	1	1	1	1	1	1	1	1	1	1	1
10.	Test results deciphered without knowledge of the control method results	1	1	1	1	1	1	1	1	1	1	1	1
11.	Control method results deciphered without knowledge of the test method results	1	1	1	1	1	1	1	1	1	1	1	1
12.	Intermediate test results reported	0	0	0	1	0	0	0	0	0	0	0	1
13.	Withdrawal from the study explained	1	0	0	0	1	0	1	0	0	0	0	0
	Total	12	10	10	9	12	10	11	11	10	9	10	10

A score of 1 was assigned for “yes” answers and a score of 0 for “no” or “unclear” answers. The maximum score achievable was 13, indicating a low risk of bias.

**Table 3:** Reasons for exclusion.

Author	Reason for Exclusion
Mennito et al. [31]	Study performed on cadaveric maxilla.
Gan et al. [14]	A completely dentulous arch was studied.
Deferm et al. [32]	Interobserver validity was studied.
Peroz et al. [33]	Oral-health-related quality of life was evaluated.
Tasaka et al. [34]	Interoperator validity was assessed.
Schimmel et al. [35]	Analyzed the influence of operator’s experience on accuracy.
Mai et al. [36]	Segmental scan and merge methods were studied.
Kontis et al. [37]	Compared intraoral scans with laboratory scan of impressions and casts.
Ender et al. [38]	Complete dentition was present.
Tao et al. [39]	Accuracy comparison with and without resin markers.
Stefanelli et al. [40]	Compared scanning strategy.
Passos et al. [41]	Different intraoral scanners are compared.
Li et al. [42]	Compared the accuracy by using PEEK based scanning aid.
Baghani et al. [43]	Completely dentulous arch studied.
Gutmacher et al. [44]	Study performed on cadaveric maxilla.

**Table 4:** Characteristics of included clinical studies.

Author Year	Country	Sample Size	Intra Oral Scanner	Laboratory Scanner	Jaws	Conventional Impression Type	Scanned Surface	Accuracy Evaluation Method	Aligning Software	Mean Difference (mm)	Mean RMS and Standard Deviation (mm)
D’Arienzo et al., 2018 [20]	Italy	4	Trios 3	NR	Maxilla	Dental cast obtained from an alginate impression.	Complete edentulous jaw.	Superimposition against 3D model obtained from lab scanner.	3D Reshaper 2017	0.219 to 0.347	NR
Chebib et al., 2019 [21]	Switzerland	12	Trios 3	Iscan D103i (Imetric 3D)	Maxilla	ZOE impression (reference scan). Alginate PVS. PVS relined with ZOE (PVSM).	Complete impression surface and five different areas. Midpalatal raphe, peripheral border, inner seal, residual ridge, PPS	Superimposition against 3D model obtained from lab scanner.	Geomagic Control X64	NR	0.70 ± 0.18
Jung et al., 2019 [22]	Republic of Korea	5	CS3500 Carestream	D700, 3Shape	Maxilla and mandible	Dental cast obtained from border-molded PVS impression.	maxilla. Midpalatal raphe, hard palate, residual ridge, soft palate. Mandible: residual ridge, buccal shelf.	Superimposition against 3D model obtained from lab scanner.	Geomagic control 2014	Maxilla—0.09 ± 0.08 Mandible—0.04 ± 0.05	NR
Lo Russo et al., 2020 [23]	Italy	10 maxilla and 10 mandibles	Trios 3	NR	Maxilla and mandible	Polysulfide impression.	Complete edentulous jaw.	Superimposition against 3D model obtained from IOS.	Geomagic wrap 2017	Maxilla—0.11 ± 0.09 Mandibular—0.26 ± 0.29 (Trimmed scans) Maxilla—0.03 ± 0.03 Mandibular—0.02 ± 0.07	NR
Hack et al., 2020 [24]	USA	27 maxilla and 5 mandibles	Lava COS True Definition (3M ESPE)	D700 version 2013 3Shape	Maxilla and mandible	Border-molded PVS impression, stone cast obtained from an impression.	Complete edentulous jaws.	Conventional impressions and the resulting stone casts were digitized and superimposed over the optical impressions.	Geomagic Qualify 2013	Overall—0.363 ± 0.143 Maxilla—0.308 ± 0.050 Mandible—0.532 ± 0.119	NR
Kalberer et al., 2020 [25]	Switzerland	12	Trios 3	Iscan D103i (Imetric 3D)	Maxilla	Border-molded ZOE impression. Alginate PVS. PVS relined with ZOE.	Anterior region, buccal region, and PPS region.	Three selected reference points to superimpose the impression. Only border extension (vertical) and seal (horizontal) were assessed.	Custom-made 3D comparison software	NR	Overall vertical discrepancy 1.95 ± 0.76 Overall horizontal discrepancy 2.23 ± 0.55
Chebib et al., 2022 [26]	Switzerland	20	Trios 3	Iscan D103i (Imetric 3D)	Maxilla	Scan of definitive cast obtained from border-molded ZOE impression.	Complete edentulous jaws.	Superimposition against 3D model obtained from lab scanner.	Geomagic control X 2020	NR	0.45 ± 0.11
Al hamad 2023 [27]	Jordan	21	Trios 4	Ceramill^®^ map400	Maxilla and mandible	Border-molded PVS impression.	Complete edentulous jaws.	Superimposition against 3D model obtained from lab scanner.	Geomagic Control X; 2020	NR	Maxillary 0.92 ± 0.24 Mandibular 1.38 ± 0.29

PVS: polyvinyl-siloxane, IOS: intraoral scanner, ZOE: zinc oxide eugenol impression material, RMS: root mean square deviation. PPS: posterior palatal seal, NR: not reported.

**Table 5:** Characteristics of included in vitro studies.

Author Year	Country	Sample Size (Per Scanner)	Scanner	Reference Scanner	Jaws	Surface Scanned	Accuracy Evaluation Method	Aligning Software	Result (Values in µm)		
Patzelt et al., 2013 [13]	USA	20	1. CEREC AC Bluecam 2. Lava COS 3. iTero, 4. Zfx IntraScan	Activity 101, smart Optics	Maxilla and mandible	Complete edentulous jaw.	Superimposition against 3D model obtained from laboratory scanner.	Geomagic Qualify 2012	Maxillary	Trueness	Precision
									CEREC AC Bluecam	591.8	332.4
									Lava COS	52.9	30.8
									iTero (3S)	144.2	178.5
									iTero (DW)	139.5	166.8
									Zfx IntraScan	283.8	425.3
									Mandibular	Trueness	Precision
									CEREC AC Bluecam	558.4	698.0
									Lava COS	44.1	21.6
									iTero (3S)	191.5	197.9
									iTero (DW217.3)	154.7	217.3
									Zfx IntraScan	253.8	319.4
Braian and Wennerberg 2019 [28]	Sweden	15	1. Omnicam 2. Itero 3. Planmeca 4. Carestream CS3600 5. TRIOS 3	NR	Mandible	Complete edentulous jaw.	Measurement of distance between markers in edentulous arch and comparison with the reference data.	No aligning software used		Trueness	Precision
									Omnicam	193	299
									Itero	81	85
									Planmeca	145	441
									CarestreamCS3600	181	247
									TRIOS 3	36	94
Osnes et al., 2020 [29]	Italy	5	1. True Definition 2. Planmeca 3. Omnicam 4. Dental wings 5. Trios 3 6. Aadva	NR	Maxilla	Complete edentulous jaws.	Superimposition of 3D data within each group to assess precision.	Custom-made software	Mean deviations		
									True Definition	250	
									Planmeca	870	
									Omnicam	320	
									Dental wings	970	
									Trios 3	260	
									Aadva	30	
Zarone et al., 2020 [30]	Italy	10	Trios 3	ATOS core 80	Maxilla	Complete edentulous, smooth, and wrinkled model.	Superimposition against 3D model obtained from lab scanner.	Geomagic Control X		Trueness	Precision
									WT/BP	48.7	46.7
									WT/SS	65.9	53.6
									WT/PB	109.7	90
									ST/BP	48.1	46
									ST/SS	56.4	76
									ST/PB	61.1	52.9

BP: buccopalatal technique; PB: palatobuccal technique; SS: S-shaped technique; ST: smooth typodont; WT: wrinkled typodont; NR: not reported.

## Data Availability

Not applicable.
